# Multifocal nodular lesions in fatty liver mimicking neoplastic disease: a case report

**DOI:** 10.2144/fsoa-2022-0084

**Published:** 2023-03-28

**Authors:** Manel Moalla, Amal Khsiba, Moufida Mahmoudi, Khaled Bouzaidi, Emna Chelbi, Asma Ben Mohamed, Manel Yakoubi, Mouna Medhioub, Lamine Hamzaoui, Mohamed Moussadek Azzouz

**Affiliations:** 1 Department of Gastroenterology, Mohamed Taher Maamouri Hospital, Nabeul, Tunisia; 2 Department of Radiology, Mohamed Taher Maamouri Hospital, Nabeul, Tunisia; 3 Department of Pathology, Mohamed Taher Maamouri Hospital, Nabeul, Tunisia

**Keywords:** fatty liver, liver biopsy, metastasis, NASH, steatosis

## Abstract

Usually, fatty hepatic infiltration is diffuse and homogeneous. However, in some cases, it can be localized simulating benign or malignant tumors. We present a case of a 61-year-old female patient with family history of malignancy: sister with lung cancer, an other sister with colon cancer and a mother with breast cancer; who presented with multiple hepatic nodules at the ultrasonography images. CT scan and MRI were not sufficient to pose a certain diagnosis which was later confirmed by liver biopsy.

## Plain language summary

In some cases, fatty hepatic infiltration may simulate benign or malignant tumors. We present a case of a 61-year-old female patient with family history of malignancy who presented with multiple hepatic nodules at the ultrasonography images. CT scan and MRI were not sufficient to pose a certain diagnosis which was later confirmed by liver biopsy.

Liver steatosis is a well defined entity characterized by abnormal accumulation of fat in the liver tissue. Its prevalence is increasing affecting currently 25–30% of the general population [1]. Usually, fatty hepatic infiltration is diffuse and homogeneous. However, in some cases, it can be localized simulating benign tumors such as hepatic adenoma, atypical hepatocellular neoplasm or angioma or malignant ones [2]. It may lead to diagnostic confusion especially when there are multiple lesions requiring more imaging investigations and in some cases, liver biopsy. Recently, we dealt with a patient raising this diagnostic problem.

## Case report

**Figure 1.**
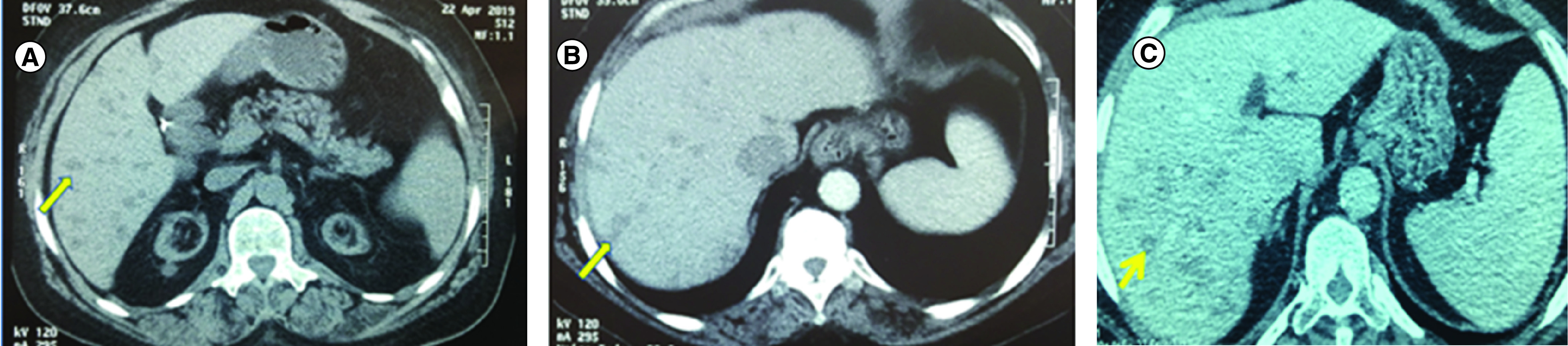
CT scan images in different phases. **(A)** Non-contrast enhanced CT scan showing multiple hypodense lesions. **(B)** Same findings on contrast enhanced CT scan in the arterial phase. **(C)** Same findings on contrast enhanced CT scan in the portal phase.

A 61-year-old female patient with medical history of arterial hypertension, hypothyroidism, end-stage renal failure on chronic hemodialysis and cholecystectomy presented to our gastroenterology department with a 3 month hypogastric pain. She had family history of a sister with lung cancer at the age of 64-years old, an other sister with colon cancer at 58-years old and a mother with breast cancer at an age above 70. We could not have details about the histological types of these cancers. The clinical examination revealed a preserved well-being and a BMI at 31.7 kg/m^2^. The abdominal exam was normal. Laboratory tests showed normocytic anemia: hemoglobin at 8.9 g/dl, thrombocytopenia at 135,000 cell/mm^3^. The hepatic tests were normal. Abdominal ultrasonography revealed a 17 cm hepatomegaly with multiple nodular hyperechoic lesions that looked unvascular at the doppler ultrasound. A CT scan showed multiple centimetric liver lesions hypoattenuating compared with the adjacent liver without any enhancement suggesting metastatic lesions (Figure 1).

**Figure 2.**
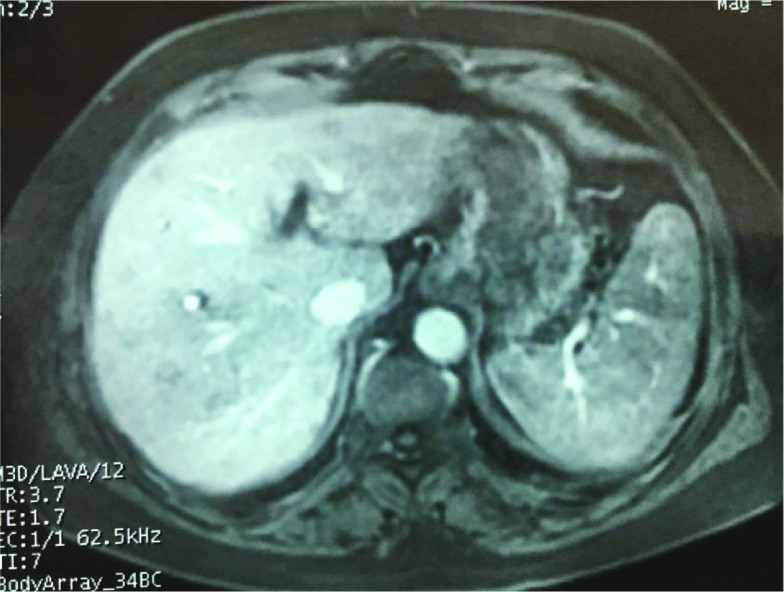
Contrast enhanced T1-weighted sequence on MRI showing very week enhancement.

An hepatic MRI was practiced showing lesions smaller than one centimeter with hyposignal on all acquisitions and very week enhacement after gadolunium injection in the arterial phase (Figure 2). Portal and tardive phases as well as fat suppression sequence showed lesions in hyposignal compared with the rest of hepatic parenchyma.

**Figure 3.**
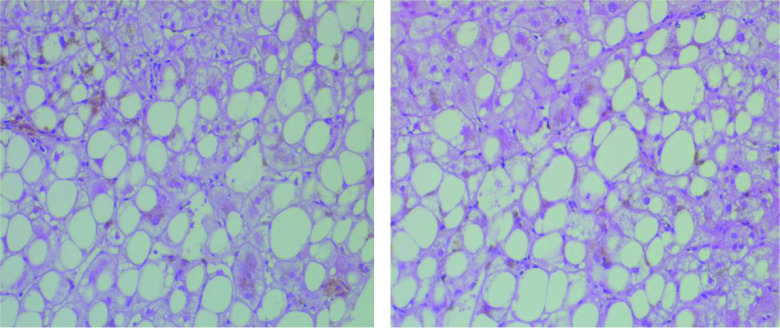
Macrovacuolar and heterogeneous steatotic foci predominant in the centrilobular and perivascular areas. Hematoxylin and eosin, ×400.

Thus, we performed an upper gastrointestinal endoscopy, a colonoscopy, a mammography and a cervical ultrasonography in order to look for a primary cancer responsible of hepatic metastases. They showed a congestive gastropathy, sigmoid diverticulitis, ACR-2 (benign) and ACR-3 (probably benign) lesions according to the American College Of Radiology and a multinodular goiter respectively. Fine needle aspiration of thyroid nodule was negative. Giving the negativity of all exams, liver biopsy was mandatory. It was practiced by an experimented radiologist using a fine needle and under scannographic control. It revealed heterogeneous fat deposits with mild inflammatory signs and ruled out malignancy (Figure 3).

## Discussion

Fatty liver is a well recognized disease defined by macrovesicular fat infiltration affecting more than 5% of the hepatocytes [3,4]. Its prevalence is in perpetual increase all over the world. It may be caused by various metabolic disorders. Nonalcoholic fatty liver disease represents the main cause. It constitutes a world wide health problem with an increasing prevalence reaching 30% of adult population in the USA [3,5]. Other metabolic disorders may lead to fatty liver such as alcohol intake, diabetes mellitus, obesity, malnutrition, parenteral nutrition, drug use, cystic fibrosis, jejunoileal bypass surgery, pregnancy and other pathologies [6]. Our patient was obese with a BMI of 31.7 kg/m^2^. The gold standard for diagnosis is liver biopsy. However, since biopsy is an invasive procedure, other criteria have been established to lead diagnosis based on ultrasonography, CT scan and MRI [7,8]. Fatty liver affects usually the liver in a homogeneous way (68%). Thus, it appears hyperechoic on ultrasonography, hypodense on non contrast enhanced CT images, hypointense on opposed phase T1 weighted MRI and on fat suppressed MRI sequence [9,10]. The diagnostic problem occurs when the fatty infiltration is not diffuse. Various patterns of fat deposition are described: geographic, subcapsular, perivascular, solitary (9%) or multifocal (22%) focal pattern [9,11]. Multifocal nodular steatosis consists of multiple, small (0.5–2 cm), fatty nodules within the liver, which are isolated or coalesce. Its physiopathology remains controversial. Insulin level and portal blood flow alteration may play a role in the development of these nodules. As this entity is uncommon and sometimes misdiagnosed as metastatic disease, a diagnosis of multinodular focal steatosis should always be taken into account in case of multiple, small fatty lesions encountered in patients with no underlying history of malignancy. In our case, fatty lesions were multifocal mimicking metastatic lesions. In these cases, MRI is the benchmark imaging tool. Fatty lesions appear hyperintense on T1-weighted and T2-weighted TSE sequences. Fat suppressed T2-weighted TSE sequence is a reliable tool to differentiate between benign multifocal hepatic steatosis from metastases [12]. As well, mass effect in CT scan or MRI can help differentiate liver metastases from focal fatty liver. Mass effect is demonstrated by displacement of vessels and loss of normal vascular branching. On CT scan, liver metastases are usually round or oval, and have CT density values close to normal parenchyma while focal fatty lesions are usually non spherical with a density close to water [13]. In the case of our patient, neither CT scan nor MRI could determine the nature of lesions. Besides, our patient had a rich family history of cancer including colon, breast and lung cancer. Some genetic mutations can be responsible of colon and breast cancer association, the most known are *BRCA1* and *BRCA2* mutations [14]. Thus, it was crucial to have a certain diagnosis in our patient and exclude cancer with certainty which led us to practice liver biopsy biopsy that showed macrovacuolar and heterogeneous steatotic foci predominant in the centrilobular and perivascular areas concluding to fat infiltration of the liver.

## Conclusion

The present case report shows that multifocal hepatic steatosis may simulate neoplastic disease, in particular in patients with underlying history of malignancy. In most of the cases, imaging tools such as CT scan and MRI are sufficient to make the diagnosis. Seldom, we are obliged to practice liver biopsy for a certain diagnosis.

**Executive summary**

- Fatty liver affects usually the liver in a homogeneous way (68%).
- Computed tomography and MRI are some times unable to distinguish between fatty lesion and hepatic tumor.
- Liver biopsy remains the gold standard for certain diagnosis of hepatic nodular lesions.
